# Development of Drug-Loaded Gelatin-Based Hydrogel Films for Impaired Wound Healing in Simulated Chronic Conditions

**DOI:** 10.3390/pharmaceutics18010043

**Published:** 2025-12-29

**Authors:** María del Carmen Morán, Alessia Cocci Grifoni, Francesca Cirisano, Michele Ferrari

**Affiliations:** 1 Departament de Bioquímica i Fisiologia, Secció de Fisiologia—Facultat de Farmàcia i Ciències de l’Alimentació, Universitat de Barcelona, Avda. Joan XXIII, 27-31, 08028 Barcelona, Spain; 2 Institut de Nanociència i Nanotecnologia—IN2UB, Universitat de Barcelona, Avda. Diagonal, 645, 08028 Barcelona, Spain; 3 CNR-ICMATE Istituto di Chimica della Materia Condensata e di Tecnologie per l’Energia, Via De Marini, 6, 16149 Genova, Italy; francesca.cirisano@cnr.it

**Keywords:** biocompatibility, contact angle, gelatin, hydrogel films, selective toxicity, vapor water transmittance rate, work of adhesion

## Abstract

**Background/Objectives**: Chronic wounds are considered a silent epidemic, affecting a significant portion of the global population and often leading to severe complications. In particular, wounds resulting from burns or trauma can give rise to squamous cell carcinoma (SCC), a form of skin cancer that arises under chronic inflammatory conditions. This study aims to develop and evaluate pH-responsive gelatin-based hydrogel films incorporating 5-fluorouracil (5-FU) for targeted treatment of SCC in chronic wound environments. **Methods**: Hydrogel films were formulated using gelatin and loaded with 5-FU. The design leveraged the pH differences between healthy skin and chronic wounds to enable stimuli-responsive drug release. The hydrofilms were characterized by evaluating their surface properties, including transparency, contact angle, and nanoscale morphology. In vitro swelling and dissolution behaviors were analyzed under varying pH conditions. Hemocompatibility was assessed through standard blood interaction assays. Cytotoxicity and selective toxicity were tested using both non-tumoral and tumoral representative skin cell lines. **Results**: The hydrogel films demonstrated pH-dependent swelling and dissolution, aligning with the neutral and basic environment of chronic wounds. Surface analysis revealed suitable transparency, wettability, and nanoscale uniformity for wound application. In vitro studies showed excellent hemocompatibility. Cytotoxicity assays confirmed good selective toxicity against the A431 skin carcinoma cell line, with minimal effects on healthy cells. **Conclusions**: The developed gelatin-based hydrogel films exhibit promising characteristics for targeted SCC therapy in chronic wounds. Their pH responsiveness, biocompatibility, and selective antitumor activity support their potential as effective and safe delivery systems. This platform may offer a novel therapeutic approach for managing malignancies arising in non-healing wound environments.

## 1. Introduction

Wound healing is a complex physiological process that progresses through the phases of hemostasis, inflammation, proliferation, and remodeling. Chronic wounds, such as diabetic foot ulcers, pressure ulcers, and venous leg ulcers, often become arrested in the inflammatory phase, leading to persistent tissue damage and delayed healing [1,2]. These wounds typically exhibit an alkaline microenvironment (pH 7.2–8.9), unlike healthy skin and acute wounds, which maintain a mildly acidic pH (~5.5). This elevated pH promotes bacterial growth and ECM degradation, further impairing tissue regeneration [3].

To improve chronic wound treatment, there is increasing interest in advanced dressings that actively support healing. Gelatin, a denatured form of collagen, is widely used due to its biocompatibility, biodegradability, low immunogenicity, and ECM-like properties that favor cell adhesion and proliferation [4,5]. Its properties can be adjusted through crosslinking or incorporation of therapeutic agents to enhance performance [6]. Notably, gelatin exhibits pH-responsive behavior, with swelling, degradation, and drug-release kinetics influenced by environmental pH [7].

Recent advancements have highlighted the potential of gelatin-based nanoparticles as drug-delivery systems for chronic wound treatment. Their nanoscale size, high surface area, and tunable surface chemistry enable the encapsulation and controlled release of bioactive compounds such as antimicrobial peptides, antioxidants, and growth factors [8,9]. Studies carried out by the author of the present paper have shown that gelatin nanoparticles can be tailored to respond to various biochemical triggers, including pH, oxidative stress, and hyperglycemia, to deliver therapeutic agents with precision [10,11].

Despite their promise, gelatin-based nanoparticles are colloidal systems and typically require a supporting scaffold or carrier for effective topical application, limiting their direct usability as dressings. In contrast, gelatin-based hydrogel films provide a solid or semi-solid matrix that can be directly applied to the wound bed. These systems maintain a moist environment, enable gas exchange, and function as both physical barriers and drug-delivery platforms. Unlike nanoparticles, hydrogels form cohesive, macroscopic structures that remain localized at the application site without the need for additional carriers [12]. Furthermore, hydrogels generally exhibit slower degradation kinetics and improved mechanical integrity [13].

Gelatin-based hydrogels in thin-film form offer additional benefits in comparison with their nanoparticle counterparts. These hydrated, semi-permeable polymer membranes maintain a moist healing environment, allow for gas exchange, and serve as protective barriers against external contaminants, all while facilitating the controlled release of embedded therapeutics [12]. However, their performance under the biochemically hostile conditions of chronic wounds, marked by elevated pH, remains a key challenge for clinical application [13].

In this work, two types of gelatin-based hydrofilms were prepared by the solvent casting method: (i) pure gelatin type A (GA) films with three different gel strength values and (ii) blended films containing gelatin type A (GA) and type B (GB), with two different gel strength values. The design leveraged the pH differences between healthy skin and chronic wounds to enable stimuli-responsive drug release from these hydrogel films considering the isoelectric point (pI) of GB (between pH 4.8 and 5.2) and GA (ranged between 7 and 9 and linked to the Bloom value) [14]. The development of squamous cell carcinoma (SCC) has been reported in chronic wounds arising from burns, trauma, ultraviolet (UV) and ionizing radiation, chronic immunosuppression, diabetes, and other long-standing inflammatory conditions [15]. Interestingly, previous works carried out in our lab have demonstrated the successful encapsulation of 5-fluorouracil (5-FU) into gelatin-based nanocarriers and delivery under simulated chronic wound conditions and SCC malignancy [10]. The resulting hydrofilms were characterized by evaluating their surface properties, including transparency, wettability, and nanoscale morphology. In vitro swelling and dissolution behaviors were analyzed under varying pH conditions. The biocompatibility was assessed through the interaction with red blood cells, and the cytotoxicity and selective toxicity were tested using both non-tumoral 3T3 fibroblasts and HaCaT keratinocytes with an SCC representative cell line (A431 cell line). The developed gelatin-based hydrogel films, especially the blended systems, exhibit promising characteristics for targeted SCC therapy in chronic wounds. Their pH responsiveness, biocompatibility, and selective antitumor activity support their potential as effective and safe delivery systems for managing SCC arising in simulated non-healing wound environments.

## 2. Materials and Methods

### 2.1. Materials

Gelatin from bovine skin, gelatin type B, with gel strength 75 (GB75) and 225 (GB225) Bloom values; gelatin from porcine skin, gelatin type A with gel strength values ranging between 100 (GA100), 175 (GA175), and 300 (GA300) Bloom values; and 5-fluorouracil (5-FU) were purchased from Sigma (St. Louis, MO, USA) and used as received. In addition, 100% pure glycerin (EDDA PHARMA (Madrid, Spain)) was obtained from a local pharmacy and used as received.

Dulbecco’s Modified Eagle’s Medium (DMEM), fetal bovine serum (FBS), l-glutamine solution (200 mM), penicillin–streptomycin solution (10,000 U/mL penicillin and 10 mg/mL streptomycin), phosphate-buffered saline (PBS), and trypsin–EDTA solution (170,000 U/L trypsin and 0.2 g/L EDTA) were purchased from Corning (Manassas, VA, USA). 2,5-Diphenyl-3,-(4,5-dimethyl-2-thiazolyl) tetrazolium bromide (MTT), Neutral Red Uptake (NRU), and dimethylsulfoxide (DMSO) were obtained from Sigma–Aldrich (St. Louis, MO, USA). The 75 cm^2^ flasks and 96-well cell culture plates were obtained from TPP (Trasadingen, Switzerland). All other reagents were of analytical grade.

### 2.2. Methods

#### 2.2.1. Preparation of Gelatin-Based Hydrogel Films

Three types of hydrogel films were produced by solvent casting: pure gelatin type A systems (GA) with different gel strength values (100, 175, and 300 g Blooms) and mixed systems containing both gelatin type A and gelatin type B (GB), either 75 g Blooms (GB75) or 225 g Blooms (GB225). The gelatin powder was hydrated in PBS pH 5.5 (10% *w*/*v*) at room temperature and then heated at 75 °C at 450 rpm until total dissolution. Then, pure glycerin (30% *w*/*w* on a dry gelatin basis) was added to the gelatin solution. The solution obtained was mixed with the 5-FU solution (0.5% *w*/*w* on a dry gelatin basis) and stirred for 5 min. The suspensions (1 mL) were then cast on glass plates (2.7 cm diameter) and left to dry at room temperature for 48 h to obtain the gelatin-based films. Gelatin films in the absence of 5-FU were prepared at the same concentrations described above for comparison. Samples were conditioned at 10% humidity at 20 °C for at least 24 h prior to testing. When needed, films were cut into discs of different diameters, according to the type of experiment to be performed, using an EO sterilized skin punch (5–6 mm, OT14012, Yiwu Hongtu Handicraft, China).

#### 2.2.2. Physicochemical Characterization of the Gelatin-Based Hydrogel Films

- Surface characterization of the films

The structural characterization of the prepared hydrogels was examined by using 3D confocal and interferometric profilometry (Sensofar S-NEOX, Terrassa, Spain). Because of its quick and non-destructive use and characterization in compliance with ISO 25178 [16], 3D profilometry was selected to enable a large scanned surface. Prior to the analyses, the films were incubated at 10% humidity for 24 h. Confocal mode was used to analyze the morphology and homogeneity of the films as a function of the imposed compositions. Films were scanned and the corresponding profiles analyzed with SensoSCAN software 5.3 version (Sensofar, Terrassa, Spain). Morphological parameters such as thickness and surface roughness were also collected and analyzed. The thickness of the film was obtained by cutting the sample and performing measurements at three random positions on each film to obtain the mean thickness, reported with standard deviation. Surface roughness was obtained taking at least three measurements at different points on the sample.

- Optical properties of the films

To evaluate the UV properties and transparency of the produced gelatin, optical transmittance (%T) in the range between 280 nm and 700 nm was measured at room temperature using a UV-VIS spectrometer (Ocean Optics Flame Spectrometer, Halma Company, Amersham, UK).

- Water vapor transmission rate (WVTR)

A custom chamber (dimensions 16 × 16 × 9 cm) was designed and fabricated in our laboratory to provide precise environmental control. Film discs of 2.7 cm diameter were placed in the chamber at 10% humidity for 24 h. Once the samples were weighed, the samples were incubated in the chamber at 99% humidity for 1, 2, 3, and 7 days. Water vapor transmission rate (WVTR) was calculated by the following equation:

WVTR (g/m^2^ × day) = G/t. A (1)

where G is the change in weight (g), t is time, and A is the test area (mm^2^), which corresponded to 5.73 × 10^−4^ mm^2^.

- Water contact angle and work of adhesion

The wetting behavior of the films as a function of the imposed compositions was studied using the static sessile drop method. For the water contact angle measurements, the films were exposed to either 10% or 65% relative humidity. A drop of Milli-Q water (Milli Q Ultrapure water, EQ 7000) (Milli-Pore, Burlington, MA, USA) was placed on the film surface, and the shape of the droplet was recorded at zero and twenty seconds to investigate wetting behavior over time. Images were digitized and were analyzed with an image processor (Image J (https://imagej.net/ij/) bundled with 64-bit Java 8). Afterwards, the average of three readings taken on each droplet was used to evaluate the water contact angle (WCA). The work of adhesion (WoA) parameter was also determined considering the contact angle measurements obtained through the sessile drop analysis using the following equation:

WoA (mN/m) = γ_L_ (1 + cos θ) (2)

where γ_L_ is the surface tension of pure water, which corresponded to 72.8 mN/m at room temperature, and θ is the corresponding water contact angle.

- Swelling and dissolution behavior

Studies were performed in PBS at various pH (5.5, 7.4, and 9.0) to determine their pH-responsive behavior. The sliced hydrogel films were weighed (approximately 100 mg) and exposed to the dissolution media (1 mL) at an agitation rate of 30 rpm using a PMR-30 Mini Rocker-Shaker (Grant Bio, dDBiolab, Barcelona, Spain). At defined time intervals, the corresponding media was carefully removed, and the hydrogel films were weighed. Then, new solution was added to maintain a clean environment. This procedure was repeated until the films were completely dissolved. The data were then transformed into a percentage of swelling (SW) using the following equation:

SW (%) = (Wf − Wi)/Wi × 100 (3)

where Wi = hydrogel initial weight and Wt = hydrogel at the time t.

- Kinetics of 5-FU release

Simultaneously with the studies of swelling/dissolution behavior, 5-FU release studies were carried out. Hence, at defined time intervals, the supernatant was collected, and films were resuspended in fresh solutions. 5-FU released into the supernatant solutions was quantified by spectrophotometry at 267 nm using a nanophotometer (NanoPhotometerTM, Implen GmgH, München, Germany). The concentration of free 5-FU in the solution was determined by interpolation from the calibration curve.

#### 2.2.3. Biological Characterization of the Gelatin-Based Hydrogel Films

- Hemocompatibility studies

1. 1. Obtention and extraction of the erythrocytes

Samples of human blood were obtained from the Banc de Sang i Teixits de Barcelona (Blood and Tissue Bank) from the Catalan Department of Health. Blood was deposited in tubes with anticoagulant EDTA-K3. The blood samples were centrifuged at 3000 rpm at 4 °C for 10 min (Megafuge 2.0 R. Heraeus Instruments, Hanau, Germany). The resulting supernatant containing plasma was extracted with a Pasteur pipette. Then, the blood samples were washed with PBS pH 7.4. This procedure was repeated three times to remove residual leukocytes and platelets and to concentrate the erythrocytes. Then, the erythrocyte suspension was diluted (1:1) in PBS pH 7.4 to obtain a suitable erythrocyte suspension to work with (cell density of 8 × 10^9^ cell/mL).

2. Hemolysis assay

The hemolysis assay determines the capability of gelatin-based films to induce lysis activity in the erythrocyte membrane. Film discs of 6 mm diameter were placed in polystyrene tubes, and an aliquot of 25 μL of erythrocyte suspensions was added to each tube. The final volume (1 mL) was achieved by addition of PBS at either pH 5.5., 7.4, or 9.0. The tubes were incubated at room temperature for 10 min in shaking conditions (MiniLab Roller, Labnet International, NJ, USA). After that, the tubes were centrifuged at 10.000 rpm for 5 min (Heraeus Biofuge pico, Heraeus Instruments, Hanau, Germant). The degree of hemolysis was determined by comparing absorbance at 540 nm (Shimadzu UV-160A, Shimadzu Corporation, Kyoto, Japan) of the supernatants with those of the control samples hemolyzed with distilled water. Negative control was obtained by incubating an aliquot of 25 μL of the erythrocyte suspension with PBS pH 7.4.

- Cytotoxicity studies

1. 1. Cell cultures

The murine Swiss albino fibroblast (3T3), the immortal human keratinocyte (HaCaT), and the squamous cell carcinoma (A431) were obtained from Celltec UB. Cells were grown in DMEM medium (4.5 g/L glucose) supplemented with 10% (*v*/*v*) FBS, 2 mM l-glutamine, 100 U/mL penicillin, and 100 μg/mL streptomycin at 37 °C, 5% CO_2_. Cells were routinely cultured in 75 cm^2^ culture flasks and were trypsinized using trypsin–EDTA when the cells reached approximately 80% confluence.

2. Cell viability assays

The 3T3 and HaCaT cells (1 × 10^5^ cells/mL) and A431 cells (5 × 10^4^ cells/mL) were grown at the defined densities in the central 60 wells of a 96-well plate. Cells were incubated for 24 h under 5% CO_2_ at 37 °C. The spent medium was then removed, and cells were incubated for 24 h with film discs of 5 mm diameter in DMEM medium supplemented with 5% FBS (100 μL). The influence of pH representative of chronic wound conditions was simulated by incubating the films previously treated with suitable volumes of PBS pH 7.4 and PBS pH 9.0.

Using the MTT assay, living cells were found to reduce the yellow tetrazolium salt, 2,5-Diphenyl-3,-(4,5-dimethyl-2-thiazolyl) tetrazolium bromide (MTT), to insoluble purple formazan crystals. After the cells were incubated for 24 h with the corresponding systems, the medium was removed, and 100 μL of MTT in PBS (5 mg/mL) diluted 1:10 in culture medium in the absence of phenol red and FBS was added to the cells. The plates were incubated for 3 h, after which the medium was removed. After that, 100 μL of DMSO (Sigma–Aldrich, St. Louis, MO, USA) were added to each well to dissolve the formazan salt. Plates were placed in a microtiter-plate shaker for 5 min at room temperature to ensure the total dissolution. A Bio-Rad 550 microplate reader was used to determine the absorbance at 550 nm of the resulting solutions. The effect of the assayed conditions was calculated as the percentage of tetrazolium salt reduction by viable cells against the control cells (cells without any treatment).

3. Selectivity toward cancer cells

The corresponding selectivity indexes (SIs) of the different formulations at the different pHs toward the tumoral A431 cell line were calculated with the following equation:

SI = % Cell viability (non-tumoral cell line)/% Cell viability (tumoral cell line) (4)

where either 3T3 fibroblasts or HaCaT keratinocytes were used as representative skin model cell lines under non-tumoral conditions.

#### 2.2.4. Statistical Analysis

IBM SPSS Statistics 29 software (IBM, Armonk, NY, USA) was used for statistical analyses. Differences between datasets were considered by one-way analysis of variance (ANOVA) following the Scheffé post hoc tests for multiple comparisons. Differences were considered statistically significant at *p* < 0.05 (unless otherwise mentioned). Results are reported as means ± standard deviation. Significant differences are highlighted with an asterisk or other symbols.

## 3. Results

### 3.1. Preparation of Gelatin-Based Hydrogel Films

**Figure 1:**
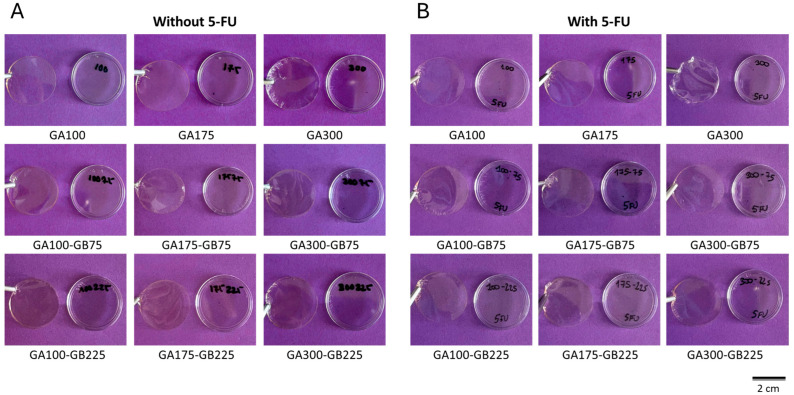
Representative images of the macroscopic morphology of the gelatin-based hydrogel films in the absence (**A**) and presence (**B**) of 5-FU.

Several gelatin-based hydrofilm formulations were prepared and optimized to obtain mechanically stable films suitable for handling and application. Hydrogel films were prepared by the solvent casting method, varying both the amount of gelatin and plasticizer in the absence of any chemical crosslinker. Trials with gelatin concentrations below 10% *w*/*v* produced solutions with insufficient viscosity for film formation and were excluded from further analysis. Among the plasticizer concentrations tested, 30% *w*/*w* glycerin (based on dry gelatin weight) provided films with appropriate flexibility and low tackiness, whereas higher amounts resulted in overly adhesive films. Representative images of the optimized 10% gelatin-based hydrofilms are shown in Figure 1, showing films in the absence (Figure 1A) and presence (Figure 1B) of 5-FU. The films were easily manageable, could be lifted with tweezers, and were selected for subsequent characterization and studies.

### 3.2. Physical and Physicochemical Characterization of the Gelatin-Based Hydrogel Films

#### 3.2.1. Surface Characterization of the Films

**Figure 2:**
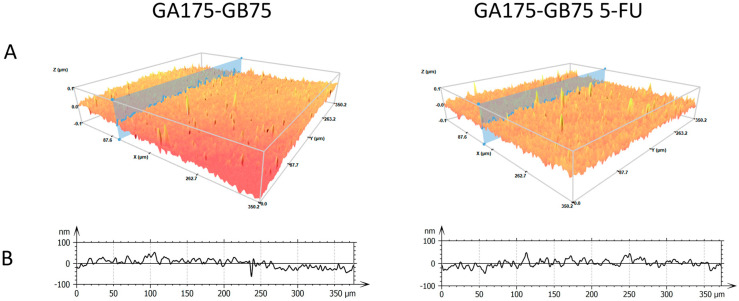
False color 3D surface topography of gelatin-based hydrofilms acquired via 3D confocal and interferometric profilometry over a 350 × 350 µm area. Color represents local height (Z, in nm), with warmer colors indicating higher features. The 3D surface maps (**A**) are accompanied by corresponding height profiles along the indicated line (**B**). GA175-GB75 average roughness, Sa = 29 nm; GA175-GB75 5-FU, average roughness, Sa = 32 nm.

The gelatin-based hydrogel films, with or without 5-FU, were analyzed with a focus on surface morphology and roughness (Sa). Topographical assessment by 3D confocal and interferometric profilometry revealed homogeneous, flat surfaces free of corpuscles or defects, as exemplified in Figure 2A for GA175-GB75 films in the absence and presence of 5-FU. Analysis of the corresponding surface profiles (Figure 2B) showed that roughness values were in the nanometric range (10–90 nm). No significant differences in morphology or roughness were observed between unloaded and 5-FU-loaded films, indicating that drug incorporation does not alter the physical characteristics of the matrices. Furthermore, the thickness of the samples was measured using the 3D profilometer. Each sample exhibited a uniform thickness, with film thickness values ranging from 130 to 230 µm (Table 1). No trends or differences were observed that could be attributed to the film composition.

#### 3.2.2. Optical Properties of the Films

The optical properties of films play a crucial role in determining both their performance and their suitability for medical applications. Transmittance (%T) data are particularly important because they reveal key aspects of the material’s behavior.

Many articles, to calculate transparency, consider the empirical formula suggested by [17], (Transparency = log(%T_600_)/x), which is correlated with the film thickness (x). However, studying the state of the art, Zhao et al. [18] found by an experimental approach that for highly transparent films, as in our case, transparency should not be expressed as a function of the film thickness, but exclusively as T% measured at 600 nm. Moreover, films that exhibit strong absorption in the 280–320 nm (UVB) and 320–400 nm (UVA) regions can provide effective UV-blocking protection. Table 1 presents representative %T values at 600 nm along with the corresponding transparency values. The %T values measured at 280 and 320 nm (within the UVB region) are also reported.

The optical characterization of GA-based films provides valuable insight into how composition and drug incorporation influence their light transmission behavior. As shown in Table 1, the neat GA films exhibited relatively high transmittance at 600 nm, indicating good transparency independent of the thickness. The introduction of GB into the GA matrix (GA-GB75 and GA-GB225 films) generally resulted in an increase in transmittance. The macroscopic characteristics of the films, being thin and transparent, could be useful in wound healing to monitor the tissue underneath with no need to remove the dressing, thus avoiding the damage and pain that dressing removal inflicts (Figure S1, Supplementary Materials).

The incorporation of 5-FU led to a noticeable decrease in transmittance across all formulations, especially at 600 nm, indicating that the drug reduces film transparency. This effect can be explained by increased light scattering caused by drug–polymer interactions. Despite the reduction in visible transparency, the films containing 5-FU demonstrated enhanced UV-blocking performance, as reflected by their lower T values at 280 and 320 nm (UVB). More limited reduction (T values ranged between 60% and 90%) was found for UVA (320–400 nm). This behavior is advantageous for biomedical applications, as it suggests that drug-loaded films can provide additional protection against UV radiation without significantly compromising optical clarity in the visible range.

#### 3.2.3. Water Vapor Transmission Rate (WVTR)

**Figure 3:**
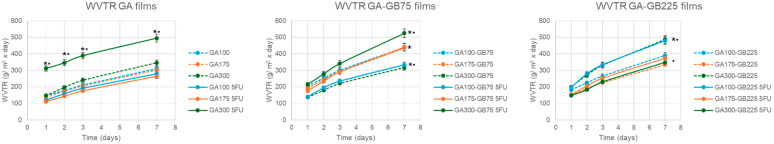
Kinetics of WVTR of the gelatin-based hydrogel films (either GA or blended with GB) in the absence and presence of 5-FU. For each condition, the results are reported as mean ± standard deviation from three independent experiments. * *p* < 0.05 indicates significant differences between values as a function of GA gel strength; • *p* < 0.05 indicates significant differences between values in the absence or presence of 5-FU.

WVTR values of the gelatin-based films were determined for 1, 2, 3, and 7 days, demonstrating an increasing trend as a function of time (Figure 3). In addition, the WVTR values seem to be mainly dependent on the GA gel strength and less dependent on the 5-FU content. In the case of pure GA systems, values ranged between 100 and 300 for GA175 and between 300 and 500 for GA300, in both cases in the presence of 5-FU. By mixing with GB75, WVRT values ranged between 150 and 300 for GB300-GB75 and between 215 and 525 for GB300-GB75 (5-FU). By mixing with GB225, WVTR values ranged between 150 and 350 for GB300-GB225 and between 200 and 500 for GB100-GB225 (5-FU).

#### 3.2.4. Water Contact Angle (WCA) and Work of Adhesion (WoA)

In wound-healing applications, WCA and WoA indicate the film’s wettability and adhesion, which affect its interaction with the wound and body fluids. Surfaces with WCA < 90° are hydrophilic, supporting fluid spreading and cell adhesion, ideal for moist wound environments. In contrast, WCA > 90° indicates hydrophobic surfaces that resist wetting and act as barrier layers [19,20]. In this work, the contact angle between a water droplet and the film surface was measured at 10% and 64% relative humidity (RH) due to their physiological relevance: 10% RH can mimic stress conditions that impact skin hydration, wound healing, and the performance of medical coatings, while 64% RH reflects more normal skin hydration and provides more stable, realistic conditions for biomaterials used on skin or mucosa.

**Figure 4:**
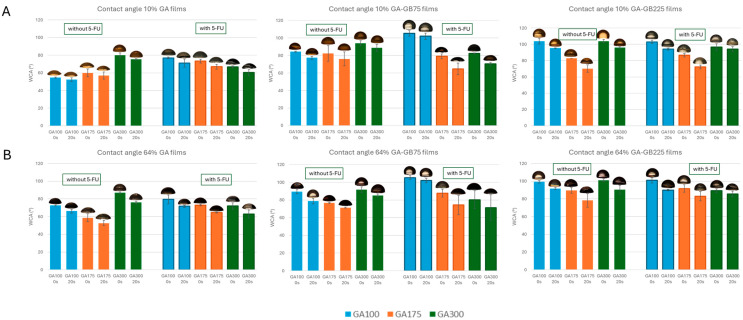
WCA values of the gelatin-based (either GA or blended with GB) in the absence and presence of 5-FU at two different RHs: 10% (**A**) and 64% (**B**). For each condition, the results are reported as mean ± standard deviation from three different drops, each with three different measurements. Representative images of the water droplets used for WCA calculations are shown at the top of each column.

Representative images of the water drop and calculated WCA values of the gelatin-based films in the absence and presence of 5-FU at two different RH values are shown in Figure 4. WCAs were determined at 0 and 20 s to assess the dynamic film wettability and liquid absorption behavior of the films.

As a general trend, pure GA films exhibited high hydrophilicity, with low initial WCA (around 46° for GA100) and a rapid decrease over 20 s (dropping to approx. 35°), indicating strong water affinity. Blending GA with GB components such as GB75 or GB225 significantly increased hydrophobicity, especially when combined with higher GA gel strength. For instance, the GA300-GB225 film showed a WCA of 110° at 0 s, decreasing only slightly to 106° at 20 s. WCA measurements showed that increasing GA gel strength (from GA100 to GA300) and blending with GB (either GB75 or GB225) enhanced the hydrophobicity of the films, as evidenced by higher initial contact angles and reduced angle decay over 20 s.

The presence of 5-FU also affects the rate of contact angle decay. Films with 5-FU generally showed a faster decrease, suggesting greater water spreading and enhanced surface wetting over time. For example, GA300-GB225 (5-FU) showed changes from 97° to 87°, compared with 112° to 107°, for the same film in the absence of 5-FU. The 5-FU incorporation significantly increases surface wettability of GA-based films by reducing both initial and dynamic contact angles. This effect is consistent across GA gel strength and blending types, indicating that 5-FU acts as a surface-active hydrophilic agent. From a functional perspective, this means that drug-loaded films may offer faster wetting and dissolution profiles, beneficial for topical delivery.

Relative humidity (RH) had a significant effect (Figure 4A,B): films conditioned at 64% RH exhibited lower contact angles and greater angle decay compared with those stored at 10% RH, indicating increased surface hydration and water affinity under humid conditions. The most water-resistant and surface-stable formulation was the 64% GA300-GB225 film, which exhibited the highest initial contact angle (approx. 112°) and maintained excellent stability over time, with only a ~5° reduction after 20 s. These results highlight the critical role of composition in tuning film wettability and support the potential use of such blends in moisture-resistant or controlled-release applications.

**Figure 5:**
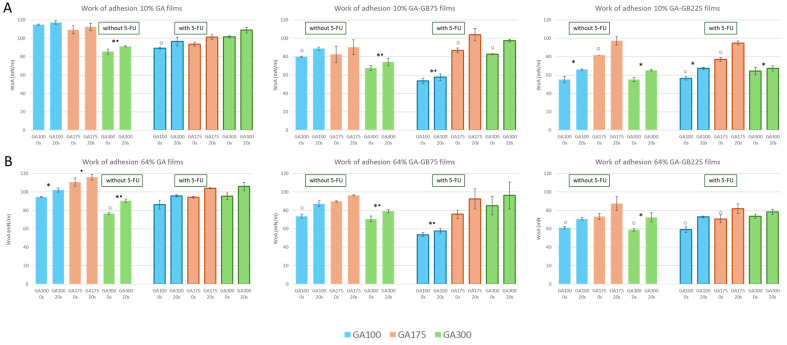
WoA values of the gelatin-based hydrogel films (either GA or blended with GB), in the absence and presence of 5-FU, at two different relative humidities: 10% (**A**) and 64% (**B**). For each condition, the results are reported as mean ± standard deviation from three different drops, each with three different measurements. * *p* < 0.05 indicates significant differences between values as a function of GA gel strength, • *p* < 0.05 indicates significant differences between values in the absence or presence of 5-FU, and ^○^ *p* < 0.05 indicates significant differences between values at 0 and 20 s.

When considering gelatin-based films for wound healing, the WoA is a key parameter that affects how well the film adheres to the wound bed, promotes healing, and remains comfortable and safe during use [21]. The WoA, which reflects the energetic cost of detaching water from the film surface, is inversely related to the water contact angle (WCA): lower contact angles generally exhibit higher WoA values due to stronger water–surface interactions. This trend is well observed in the current data (Figure 5). Pure GA films, known for their hydrophilicity, exhibit some of the highest WoA values (especially in the 10% RH), with values exceeding 110 mN/m for GA100 and GA175. However, increasing GA gel strength to GA300 slightly reduces WoA, aligning with previously observed increases in WCA. Blending GA with hydrophobic protein-based components (GB75 and GB225) reduces WoA values, particularly at higher GA gel strength, confirming enhanced hydrophobic character and lower wettability. For instance, at 65% humidity, GA300-GB225 films showed WoA values around 80–85 mN/m, while their corresponding WCA values remained high (95–100° at 0 s), indicating less water adhesion. Interestingly, 5-FU incorporation appears to slightly increase WoA in GA175-based blends, particularly in the 10% RH condition. This might reflect improved hydrogen bonding or increased film surface polarity due to the drug, which is moderately hydrophilic.

Humidity also affects adhesion: films prepared at 64% RH consistently show lower WA than those at 10% RH, likely due to denser polymer packing and surface rearrangement, which reduce available hydrophilic sites. This trend is particularly evident in GA100 and GA175 formulations, which rely heavily on hydrophilic functional groups for water interaction. In hydrophobic films, the WoA is generally less sensitive to change in humidity compared with hydrophilic films.

#### 3.2.5. Swelling/Dissolution Behavior

**Figure 6:**
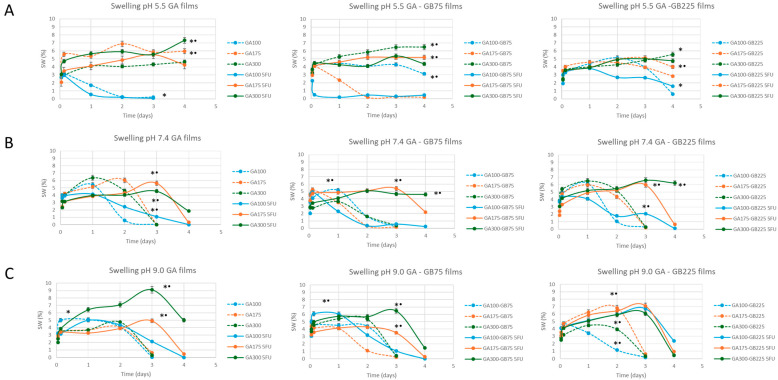
Swelling behavior of the gelatin-based hydrogel films (either GA or blended with GB), in the absence and presence of 5-FU, at representative pH of normal skin (pH 5.5 (**A**)) and pH mimicking chronic wound conditions (pH 7.4 (**B**) and pH 9.0 (**C**)). For each condition, the results are reported as mean ± standard deviation from three independent experiments, each with three technical replicates. * *p* < 0.05 indicates significant differences between values as a function of GA gel strength; • *p* < 0.05 indicates significant differences between values in the absence or presence of 5-FU.

The swelling and dissolution behaviors of GA-based films, either pure or blended with GB (GB75 and GB225) and in the absence or presence of 5-FU, were investigated under pH conditions representative of normal skin (pH 5.5) and chronic wounds (pH 7.4 and 9.0) (Figure 6). The limited swelling and long-term stability of our non-crosslinked gelatin-based hydrofilms arise primarily from the high Bloom strength of the gelatin and the controlled drying conditions used during preparation. High-Bloom gelatin forms stronger physical networks due to increased intermolecular hydrogen bonding and triple-helix content, which effectively act as a “pseudo-crosslinked” structure, providing mechanical stability and reducing water uptake. Controlled room-temperature drying further promotes dense chain entanglement, enhancing film integrity without the need for chemical or enzymatic crosslinking.

The obtained results demonstrate that these pH environments critically influence the structural integrity of the hydrogel matrix. The swelling profiles demonstrated clear pH responsiveness across all film formulations. At pH 5.5, corresponding to healthy skin (Figure 6A), swelling remained moderate for most formulations, with peak values ranging between 4% and 8%. Notably, 5-FU-loaded films showed reduced swelling capacity, suggesting that drug incorporation may densify the matrix or reduce its hydrophilicity. The GA100 (5-FU) and GA100-GB75 (5-FU) films in particular exhibited early collapse or erosion, indicating potential instability under mildly acidic conditions.

Swelling increased markedly under more alkaline conditions (pH 7.4, Figure 6B and pH 9.0, Figure 6C), consistent with the ionization of functional groups (carboxyl and amino moieties) within the gelatin network. This effect was particularly evident in films containing GB225, which achieved higher swelling ratios and maintained structural integrity for longer periods. At pH 7.4, GA100-GB75 and GA100-GB225 showed maximum swelling (8–10%). At pH 9.0, maximum swelling was observed in some 5-FU-free films (for example, GA100-GB225), suggesting structural integrity is compromised under basic conditions.

#### 3.2.6. Kinetics of 5-FU Release

**Figure 7:**
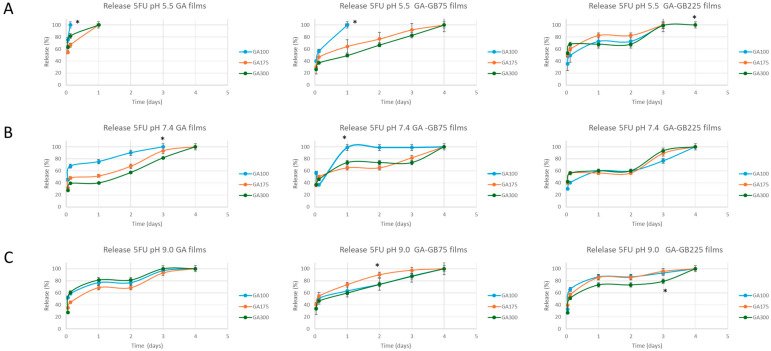
Release profiles of 5-FU from the gelatin-based hydrogel films (either GA or blended with GB) at representative pH of the normal skin (pH 5.5 (**A**)) and pH mimicking chronic wound conditions (pH 7.4 (**B**) and pH 9.0 (**C**)). For each condition, the results are reported as mean ± standard deviation from three independent experiments, each with three technical replicates. * *p* < 0.05 indicates significant differences between values as a function of GA gel strength.

The release studies revealed that drug release was strongly influenced by both pH and film composition (Figure 7). When considering the effect of GA gel strength, as a consequence of the swelling response, GA100 films may offer higher release but less control in comparison with GA300 films, which provide a more sustained release due to limited swelling. The effect of pH demonstrated that at pH 5.5 (Figure 7A), the release was generally slower and more incomplete, with cumulative release ranging from 60% to 85% over four days. GA100 films demonstrated the fastest release at this pH, likely due to their lower crosslinking density and higher swelling-driven diffusion. In contrast, GB225-containing films sustained drug release over longer durations (plateaus around 80%), likely due to a tighter network restricting molecular diffusion. The obtained results suggested that the films could promote a safe release for topical application with minimal systemic exposure in healthy skin.

As pH increased to 7.4 and 9.0, all formulations showed enhanced release rates, with several reaching near-complete release within three days. At pH 7.4 (Figure 7B), faster release kinetics were observed, especially for GA100 and GA100-GB75 (80–90% within two to three days). In the presence of GB225, a slightly more linear and delayed release was observed, promoting a pH-triggered release in a wound microenvironment. The more alkaline environment (Figure 7C) likely promotes gelatin chain relaxation and hydrolytic degradation, accelerating both swelling and drug diffusion. There was almost complete release (90–100%) within three days for all films, especially GA100 and GA100-GB75. The GA100-GB225 film provided a more gradual release, suggesting it could be used where sustained delivery is needed over several days in pathological wound settings.

### 3.3. Biological Characterization of the Gelatin-Based Hydrogel Films

#### 3.3.1. Hemocompatibility Studies

**Figure 8:**
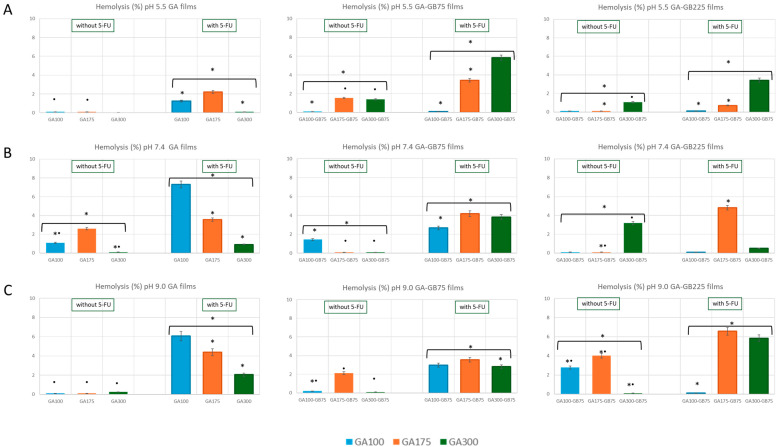
Hemolytic response of the gelatin-based hydrogel films (either GA or blended with GB) in the absence and presence of 5-FU at representative pH of the normal skin (pH 5.5 (**A**)) and pH mimicking chronic wound conditions (pH 7.4 (**B**) and pH 9.0 (**C**)). For each condition, the results are reported as mean ± standard deviation from individual experiments, each with three biological replicates. * *p* < 0.05 indicates significant differences between values as a function of GA gel strength; • *p* < 0.05 indicates significant differences between values in the absence or presence of 5-FU.

The biocompatibility of drug-delivery systems, especially those in contact with blood, is a critical parameter in biomedical applications. Hemolysis assays are widely used to evaluate the potential of materials to damage red blood cells. Hemocompatibility was assessed by evaluating the hemolysis percentage of GA-based films under various pH conditions (5.5, 7.4, and 9.0) (Figure 8).

According to ASTM F756-17 [22], materials causing less than 5% hemolysis are considered non-hemolytic and suitable for blood-contacting applications. Pure GA films demonstrated pH-sensitive hemolytic behavior: GA100 and GA175 remained below the 5% threshold across all pH levels, while GA300 exceeded this limit at physiological pH of 7.4 (approx. 8%), indicating potential cytotoxic effects at higher GA concentrations. Blending with other protein-based components such as GB75 or GB225 generally improved hemocompatibility, particularly under acidic and neutral conditions. For instance, GA300-GB225 exhibited hemolysis below 5% at both pH 5.5 and 7.4 (Figure 8A,B), although values approached the hemolytic threshold at pH 9.0 (approx. 6%, Figure 8C).

The presence of 5-FU had a clear influence on hemolysis outcomes. In most formulations, 5-FU increased hemolysis slightly, particularly in GA300-based films. For example, in the 5-FU-loaded GA300-GB225 film at pH 7.4 and 9.0, hemolysis levels were higher than their non-drug-loaded counterparts and often exceeded the 5% threshold. This increase may be attributed to the partial release of 5-FU into the surrounding medium, altering local ionic strength and surface charge, which can destabilize red blood cell membranes. Additionally, 5-FU is known to interact with membrane phospholipids and can compromise cell integrity at higher concentrations [23]. Nonetheless, in GA100 and GA175 formulations, hemolysis remained below critical levels even in the presence of 5-FU, suggesting these compositions are more compatible with drug incorporation.

All formulations showed minimal hemolysis under acidic conditions (pH 5.5, mostly < 2%), likely due to reduced polymer swelling and stronger matrix cohesion that limits drug diffusion and polymer–cell interaction. In contrast, increased hemolysis under alkaline conditions (pH 9.0), particularly in 5-FU-loaded blends, may result from elevated drug mobility and polymer ionization, exacerbating membrane disruption.

#### 3.3.2. Cytotoxicity Studies

**Figure 9:**
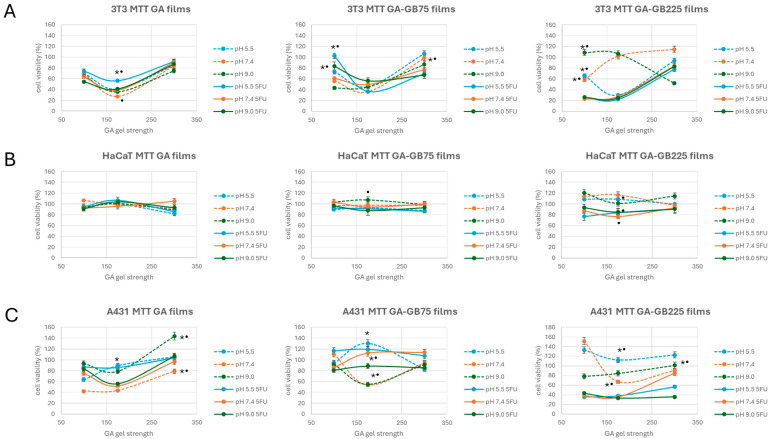
Cytotoxicity response of the gelatin-based hydrogel films (either GA or blended with GB), in the absence and presence of 5-FU, at representative pH of healthy skin (pH 5.5) and pH mimicking chronic wound conditions (pH 7.4 and pH 9.0) against 3T3 fibroblasts (**A**) and HaCaT keratinocytes (**B**) and the A431 squamous carcinoma cell line (**C**). For each condition, the results are reported as mean ± standard deviation from three independent experiments, each with three biological replicates. * *p* < 0.05 indicates significant differences between values as a function of pH values; • *p* < 0.05 indicates significant differences between values in the absence or presence of 5-FU.

To assess the biocompatibility and anticancer efficacy of the developed GA-based films, MTT assays were performed using three different cell lines: 3T3 fibroblasts and HaCaT keratinocytes (normal cells) and A431 skin carcinoma cells. This evaluation aimed to determine the influence of gel strength (either pure or blended systems), drug loading, and pH on cell viability, providing insight into the selective cytotoxic potential of the films against cancerous versus normal cells. Figure 9 shows representative results as a function of the imposed compositions.

The cytotoxicity assessment of gelatin-based films across different formulations and pH conditions revealed distinct responses between non-tumoral (3T3 and HaCaT) and tumoral (A431) cell lines. Films without 5-fluorouracil (5-FU) generally maintained high cell viability with values ranging between 80% and 100% for all three cell lines, confirming that gelatin-based films are biocompatible. The inclusion of 5-FU affects cell viability in a cell-type-dependent way. HaCaT keratinocytes exhibited stable viability (60–70%) across all conditions, consistent with their robustness and widespread use as a non-tumoral epidermal model (Figure 9B). In contrast, 3T3 fibroblasts showed reduced viability (40–60%), especially at lower GA strength, due to the faster drug release (Figure 9A). In the GB225 matrix, particularly at higher pH, moderated toxicity was observed, likely due to improved mechanical and drug-release properties associated with higher-Bloom-strength gelatin. A431 cells were highly susceptible to 5-FU-loaded films (Figure 9C), particularly at low gel strength (GA100) and at pH 7.4 and 9.0, demonstrating effective tumor-targeted cytotoxicity. Among the formulations, the GA-GB225 films showed the most favorable profile, maintaining low viability in A431 cells while preserving biocompatibility with 3T3 and HaCaT cells, even under elevated pH.

Further insights into the selective toxicity of the proposed films were deduced from the selectivity index (SI) values. Table 2 shows representative results indicating the preferential cytotoxicity of 5-FU-containing films against A431 squamous carcinoma cells compared with normal cells (3T3 fibroblasts and HaCaT keratinocytes).

The film composition as well as the cell line strongly influenced the therapeutic selectivity. GA films showed consistently lower SI values across all pH levels and drug concentrations, indicating limited ability to differentiate between cancerous and normal cells. In contrast, GA-GB composite films, particularly those with higher GB gel strength (GA-GB225), significantly improved SI. This suggests a synergistic effect of GB in modulating drug release. Moreover, increasing GA gel strength from 100 to 300 generally enhanced SI, especially in GA-GB225 films. This could be attributed to more controlled and sustained 5-FU release, leading to prolonged and preferential exposure to tumor cells while sparing normal cells.

The pH of the surrounding environment significantly affected the selectivity profiles of the formulations. At pH 5.5, SI values across most formulations were low, with several values below 1.0, indicating limited selectivity and a potential risk of cytotoxicity to normal skin cells. In contrast, pH 7.4 and 9.0 demonstrated consistently higher SI values, particularly with GA-GB composite films and in comparison with the HaCaT cell line, both keratinocytes from the same skin layer. This suggests that the alkaline microenvironment of chronic wounds enhances the selective cytotoxic effect of 5-FU on A431 cells. Notably, GA-GB225 at both GA175 and GA300 exhibited SI values > 2.0 at pH 9.0, indicating high selectivity toward tumor cells and normal cell lines (HaCaT) in this environment.

When the effect of 5-FU concentration was evaluated (1.0 mg/mL instead of 0.5 mg/mL), results demonstrated that an increase in concentration did not improve selectivity (Table S1, Supplementary Materials). While some formulations of GA-GB75 films showed dose-dependent increases in SI, particularly at lower pH values, GA-GB225 already exhibited optimal selectivity at standard conditions, with only marginal or reduced SI at the higher dose. This is especially evident at pH 9.0, where GA-GB225 at 0.5 mg/mL yielded SI values of 2.30 (3T3/A431) and 2.58 (HaCaT/A431), suggesting that lower drug doses may suffice for effective tumor targeting in alkaline wound settings, while also minimizing the risk of damage to surrounding healthy tissue. In contrast, at pH 5.5, some increases in SI at 1.0 mg/mL were observed, though values generally remained < 1.5, indicating suboptimal selectivity even at higher drug concentrations.

## 4. Discussion

The present study aims to address the critical challenge of managing SCC arising in chronic wound environments through the development of a pH-responsive gelatin-based hydrogel for targeted 5-FU delivery. The hydrogel films exhibited suitable physical properties such as thickness and transparency (Figure 1 and Table 1). Hydrogel films with approximately 200 µm thickness range are advantageous because they are thin enough for good diffusion and drug release but thick enough to provide structural integrity. Thin gelatin-based hydrogel films (100–300 µm) are often used as conformal wound contact layers or coatings that directly interact with the wound bed [5,24,25,26]. Furthermore, all prepared films show nanometric roughness and homogeneous topography, suggesting that the casting method is suitable for the preparation of gelatin films with different gel strengths and compositions (Figure 2). These findings align with recent reports that nanoscale architecture in gelatin hydrogels enhances cell–material interactions, promoting fibroblast and keratinocyte adhesion, proliferation, and migration, all key processes for wound repair [27,28,29,30]. Importantly, the incorporation of 5-FU did not alter surface morphology, maintaining smooth, defect-free films that support consistent drug delivery and reduce local irritation. Compared with crosslinked gelatin- or collagen-based films, which can exhibit variable roughness or increased stiffness, limiting conformability and drug release, our non-crosslinked high-Bloom gelatin films provide uniform nanoscale topography and mechanical stability [5,31,32].

Moreover, the assessment of the optical properties demonstrated that the combination of GB and 5-FU modulates the optical properties in a way that balances transparency with effective UVB shielding, a desirable feature for protective and therapeutic film applications (Table 1). The WVTR of a hydrogel film for wound healing applications plays a critical role in regulating moisture at the wound site. Wound dressings with low WVTR values may lead to exudate accumulation and potential maceration, whereas those with excessively high WVTR values can cause rapid dehydration and impede healing. Commercial dressings have a wide variety of WVTR values, depending on the type of wound they are made for, ranging from 90 to 3350 g/m^2^ day, tailored for the specific needs of different wound types [33]. The WVRT results obtained in this work (Figure 3), with values ranged between 100 and 500 g/m^2^ day, suggest that the proposed films may be effective to maintain a moisture balance ideal for wounds with low to moderate exudate. Low to moderate exudate could promote cell migration, stimulate angiogenesis, and prevent desiccation and scab formation [5,34].

WCA measurements revealed that surface wettability of GA-based films varied significantly with composition and humidity (Figure 4). Contact angle reduction over 20 s confirms that these films interact with water and may facilitate cell adhesion and fluid management. Pure GA films demonstrated high hydrophilicity, with GA100 showing WCA values as low as 46° at 0 s, dropping to 35° by 20 s, indicating rapid water absorption. These values fall within the desirable range for wound dressings (40–80°), which supports adequate moisture retention and exudate management without excessive adhesion [35]. Blending with GB75 or GB225 significantly increased hydrophobicity, particularly when combined with higher GA gel strength. While such hydrophobic films are unsuitable for direct wound contact, they are promising as protective outer layers or drug-release reservoirs [36]. The incorporation of 5-FU consistently reduced contact angles across all formulations, reflecting enhanced surface wettability due to the hydrophilic nature of 5-FU [37]. Additionally, conditioning films at 64% RH led to lower WCA values compared with those stored at 10% RH, suggesting increased surface hydration and softening under moist conditions. Altogether, these findings aligned well with WoA trends (Figure 4). More hydrophilic films (lower WCA) showed higher WoA, while blending with hydrophobic components or increasing humidity resulted in lower WoA values, which is desirable for wound dressings requiring reduced water affinity and better moisture barrier properties. Adhesives or films with WoA values around 30–50 mN/m, such as certain hydrocolloid- and chitosan-based films, have been reported to support clot stabilization through firm adhesion and mild tissue compression [38]. These findings correlated well with trends observed in gelatin–alginate hybrid films [26].

Non-hemolytic dressings prevent erythrocyte lysis, thereby avoiding the release of free hemoglobin and iron that can trigger oxidative stress, fuel bacterial proliferation, and exacerbate inflammation. This property is particularly valuable in chronic wounds, where delicate granulation tissue and capillary networks are easily disrupted. Clinically, the use of non-hemolytic, biocompatible dressings supports faster re-epithelialization, reduces pain and dressing-related trauma, and enhances patient comfort and adherence [39]. The hemolytic assessment indicates that GA100 and GA175 formulations were consistently hemocompatible across all pH levels, while GA300-based films may require careful pH control or blending strategies to maintain blood compatibility (Figure 8). Blending with protein-based additives like GB75 or GB225 appeared effective in mitigating hemolytic activity, especially in physiological and acidic environments.

The GA gel strength and the GB incorporation both played crucial roles in determining the functional (Figure 6 and Figure 7) and biological performance (Figure 9) of the hydrofilms. The limited swelling and long-term stability of the gelatin films are due to the combination of high Bloom strength and careful drying, which effectively produce a physically stable network without any chemical crosslinking. Pure GA films with lower gel strength (GA100) demonstrated the highest swelling capacity, attributed to their looser structure, which allows for efficient fluid exchange and moisture retention. However, this high permeability also led to rapid release of 5-FU and increased cytotoxicity, including toward normal cells (3T3 and HaCaT), indicating a need for moderation in drug delivery kinetics. Blending GA with GB (either GB75 or GB225) significantly influenced these behaviors. The GA-GB films exhibited reduced swelling compared with GA-only films, suggesting that GB incorporation could enhance structural integrity. This was particularly evident in GA-GB225 films, which showed the most controlled swelling profile through increased ionic interactions between GA and GB. Consequently, drug release was more controlled, resulting in lower cytotoxicity toward normal cells while still retaining therapeutic effects against cancer cells.

Further insights into the selective toxicity of the proposed films were deduced from the selectivity index (SI) values, which quantify the balance between cytotoxicity and therapeutic efficacy (Table 2). A higher SI value indicates that the material is substantially more toxic to cancerous cells than to normal, healthy cells, reflecting favorable therapeutic selectivity [40]. The influence of GA/GB composition on the SI values can be attributed to the combined effects of Bloom strength, polymer network density, diffusion kinetics, and pI. Higher-Bloom GA forms dense, triple-helix regions that act as pseudo-crosslinks, restricting water uptake and slowing 5-FU diffusion, while incorporation of GB increases overall polymer density, further limiting rapid drug release. The pI values of the components (GA: 7–9, depending on Bloom values; GB: 4.8–5.2) introduce additional electrostatic effects: the interaction of oppositely charged GA and GB at the pH of normal skin can stabilize the resulting hydrogels. However, at physiological or more basic pH, GA chains are near neutral or slightly negative, whereas GB chains carry more negative charges. This charge disparity modulates intra- and intermolecular interactions, affecting local network porosity and water uptake, which in turn, influence drug diffusion profiles [25,27,31]. This has been the basis of the NPs formation previously carried out in our lab [10,11]. Together with the dependence of the pI of gelatin A on gel strength, the presence of the RGD motif on the gelatin structure confers excellent properties to the targeted delivery of 5-FU to A431 cells [11]. In this work, these structural and electrostatic factors enable sustained, controlled release of 5-FU, preferentially targeting tumor cells while minimizing cytotoxicity toward normal cells, resulting in elevated SI values for GA-GB blends such as GA-GB225 hydrogel films in HaCaT/A431 systems.

Current market trends indicate a growing focus on bioactive, stimuli-responsive, and biodegradable materials in commercial hydrogel dressings, with strong emphasis on controlled drug release and long-term biocompatibility. As reported by [41], the chronic-wound segment dominated the hydrogel dressing market in 2024. In this context, the gelatin-based hydrogel films developed in this study were engineered to integrate favorable physicochemical properties together with promising preliminary biological performance, positioning them as a promising alternative to existing products.

## 5. Conclusions

The study highlights the impact of formulation design and pH on determining the therapeutic window for gelatin-based films loaded with 5-FU. The hydrogel films exhibited suitable optical and WVTR characteristics, underscoring their potential clinical relevance. Wettability and water-adhesion measurements indicated that GA films are highly hydrophobic, while blending with GB or 5-FU effectively modulates surface properties to optimize wound-management performance. The non-crosslinked films maintained sustained swelling and supported controlled release over several days, influenced by the intrinsic gel strength of GA and the incorporation of GB. Hemocompatibility testing further confirmed non-hemolytic behavior across most formulations. Among the tested formulations, GA-GB225 with higher gel strengths (175–300) under alkaline conditions (pH 9.0) demonstrated the most favorable balance, effectively targeting A431 cancer cells while preserving the viability of normal HaCaT skin cells from the same epidermal layer. These findings provide a basis for the development of pH-responsive, topical chemotherapy systems for chronic-wound-associated skin cancers.

## Figures and Tables

**Table 1:** Physical and physicochemical values related to films’ composition. Values in parentheses correspond to samples including 5-FU.

Films	GA	Thickness (μm)	T_600_	T_UVB_	
				**280 nm**	**320 nm**
GA	100	181.56 (177.68)	96.36 (98.53)	19.54 (0)	69.55 (67.37)
	175	144.91 (175.15)	93.16 (92.77)	18.13 (0)	65.87 (66.48)
	300	151.46 (147.01)	99.16 (94.83)	34.37 (0)	84.93 (87.94)
GA-GB75	100	228.90 (134.81)	100.24 (96.71)	17.47 (0)	65.14 (67.16)
	175	181.38 (216.80)	99.77 (98.24)	17.91 (0)	71.00 (61.72)
	300	153.02 (229.35)	103.63 (92.34)	21.36 (0)	71.20 (62.05)
GA-GB225	100	227.70 (208.11)	95.00 (100.8)	17.75 (0)	56.30 (60.83)
	175	197.58 (138.96)	70.1 (88.73)	7.39 (0)	41.97 (58.52)
	300	207.3 (179.43)	100.64 (87.64)	17.76 (0)	68.42 (57.23)

**Table 2:** Selectivity index (SI) values against the tumoral cell line in comparison with the fibroblast (3T3) and the keratinocyte (HaCaT) cell lines.

Film	GA	3T3/A431 pH Value			HaCaT/A431 pH Value		
		**5.5**	**7.4**	**9.0**	**5.5**	**7.4**	**9.0**
GA	100	0.86	0.84	0.65	1.10	1.23	2.15
	175	0.66	0.75	0.73	1.25	1.84	1.85
	300	0.89	0.93	0.82	0.83	1.08	0.88
GA-GB75	100	0.88	0.71	1.03	0.79	1.20	1.19
	175	0.31	0.44	0.64	0.76	0.84	0.99
	300	0.64	0.68	0.79	0.81	0.88	1.09
GA-GB225	100	0.72	0.60	0.60	2.15	2.30	2.15
	175	0.58	0.78	0.76	2.22	2.15	2.58
	300	1.37	0.98	2.33	1.61	1.09	2.54

## Data Availability

The original contributions presented in this study are included in the article/Supplementary Material. Further inquiries can be directed to the corresponding authors.

## Supplementary Materials

The following supporting information can be downloaded at: https://www.mdpi.com/article/10.3390/pharmaceutics18010043/s1, Figure S1: Representative images of the macroscopic morphology of the gelatin-based hydrogel films without and without 5-FU; Table S1: Selectivity index (SI) values at 5-FU equal to 1.0 mg/mL.
