# Supplementary material for: Development of Drug-Loaded Gelatin-Based Hydrogel Films for Impaired Wound Healing in Simulated Chronic Conditions
Source: Pharmaceutics. 2025 Dec 29;18(1):43. doi: 10.3390/pharmaceutics18010043 (PMC12845303; doi:10.3390/pharmaceutics18010043)
Supplement: Supplementary file 1 [file pharmaceutics-18-00043-s001.zip › pharmaceutics-3990293-supplementary.pdf]

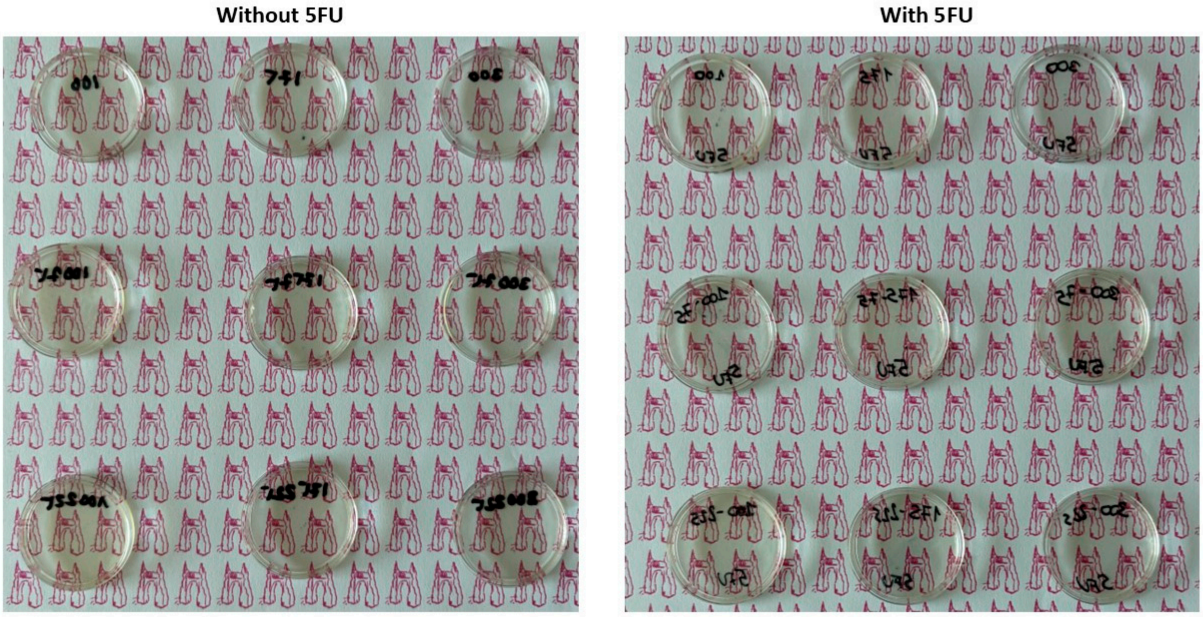


**Figure S1.** Representative images of the macroscopic morphology of the gelatin-based hydrogels without and without 5-FU.

**Table S1.** Selectivity index (SI) values at 5-FU equal to 1.0 mg/mL.

| Films | GA | 3T3/A431 pH value | | | HaCaT/A431 pH value | | |
|---|---|---|---|---|---|---|---|
| | | **5.5** | **7.4** | **9.0** | **5.5** | **7.4** | **9.0** |
| GA | 100 | 0.78 | 0.87 | 0.94 | 1.00 | 1.50 | 1.07 |
| | 175 | 1.316 | 1.34 | 0.90 | 1.16 | 2.03 | 1.11 |
| | 300 | 1.56 | 1.10 | 1.81 | 1.01 | 0.95 | 1.30 |
| GA-GB75 | 100 | 1.17 | 2.24 | 4.42 | 1.26 | 2.60 | 1.56 |
| | 175 | 2.23 | 2.68 | 1.98 | 1.94 | 2.13 | 1.44 |
| | 300 | 1.74 | 3.80 | 2.79 | 1.08 | 1.89 | 1.68 |
| GA-GB225 | 100 | 1.24 | 1.02 | 1.29 | 1.32 | 1.43 | 1.65 |
| | 175 | 1.64 | 2.10 | 3.43 | 1.63 | 1.50 | 2.15 |
| | 300 | 1.46 | 0.74 | 0.76 | 1.36 | 0.75 | 0.92 |
